# Percutaneous transhepatic venous access for hemodialysis: a single-center experience with a rescue access

**DOI:** 10.1590/2175-8239-JBN-2024-0225en

**Published:** 2026-01-30

**Authors:** Mariana Sousa Freitas, Amanda Meyer Luz, Ademar Regueira

**Affiliations:** 1 Centro Hospitalar de Trás-os-Montes e Alto Douro, Serviço de Nefrologia, Vila Real, Portugal.; 2 Hospital Regional Hans Dieter Schmidt, Serviço de Nefrologia, Joinville, SC, Brazil.; 3 Fundação Pró-Rim, Joinville, SC, Brazil.; 4 Hospital Municipal São José, Serviço de Nefrologia, Joinville, SC, Brazil.; 5 Hospital Municipal São José, Serviço de Cirurgia Vascular, Joinville, SC, Brazil.

**Keywords:** Central Venous Catheters, Hemodialysis, Transhepatic catheter

## Abstract

**Introduction:**

Central venous catheters (CVC) are often the only option for hemodialysis, particularly when arteriovenous fistulas cannot be created or in urgent situations. However, the exhaustion of traditional access sites necessitates alternative approaches. This study aims to describe our center's experience with transhepatic venous access for hemodialysis, focusing on infection rates, catheter patency, and dialysis adequacy, to evaluate the feasibility of this option in patients with limited vascular access options.

**Methods:**

We conducted a retrospective study at Pro-Rim Foundation (January 2017 – February 2024) on patients with transhepatic CVC. Clinical records were reviewed for demographics, comorbidities, CVC details, dialysis adequacy, and outcomes.

**Results:**

A total of 24 longterm transhepatic CVCs were placed in 12 patients (58.3% male, mean age 55.9 years). The technical success rate was 100%, with no complications within 24 hours. Over 3615 catheter-days, thrombosis occurred at a rate of 0.30 per 100 catheterdays, and infection occurred at 0.08 per 100 catheter-days. The mean dialysis dose (eKt/V) was 1.29. Seven patients died during follow-up, with only one death related to vascular access complications. The mean primary and secondary catheter patency times were 162.9 and 204.0 days, respectively.

**Conclusion:**

Our study supports transhepatic hemodialysis catheters as a viable option for patients with no other access options, showing good long-term functionality, low infection rates, and reasonable dialysis adequacy. Thrombosis remains a significant challenge, necessitating better maintenance, monitoring, and further research to improve outcomes.

## Introduction

According to the Brazilian Dialysis Survey (BDS) of the Brazilian Society of Nephrology (BSN)^1^, in July 2023, the estimated number of patients on chronic dialysis was 157,357, which corresponds to an increase of 2.3% compared to the previous year. Among the prevalent dialysis patients, 96.2% were on hemodialysis (HD) and 3.8% on peritoneal dialysis (PD)^1^.

In order to perform HD, it is necessary to create a vascular access. The Kidney Disease Outcomes Quality Initiative (KDOQI) clinical practice guideline for vascular access recommends the arteriovenous fistula (AVF) as the preferred access, rather than the central venous catheter (CVC), for the majority of incident and prevalent HD patients^2^. This autologous vascular access requires planning and a maturation period and it cannot be created in many patients. Currently, during vascular mapping evaluations, several patients exhibit a poor vascular patrimony, either due to a lack of arteries and veins of adequate caliber–often as a result of chronic diseases such as diabetes and atherosclerosis–or due to vein damage by several punctures during hospitalizations. Furthermore, in Brazil, many patients are admitted to the emergency department with advanced-stage chronic kidney disease (CKD), requiring urgent start of dialysis and, consequently, need for CVC implantation, with AVF construction postponed to a later phase.

In July 2023, 29.0% of patients on chronic hemodialysis had a long-term CVC as vascular access, according to the BDS of the BSN^1^. The previously mentioned poor vascular patrimony and the urgent start of dialysis, without time for vascular access planning, partially justify these numbers.

Patients who cannot have an AVF or a prosthetic arteriovenous graft created must remain on hemodialysis through long-term CVC^2^. Several studies have shown the benefit of the internal jugular vein as the primary choice for placement of long-term catheters due to the ease of the technique and low complication rates^2,3,4^. However, chronic use of these catheters carries serious risks, such as infections and failure of vascular access, with possible exhaustion of traditional sites for catheter placement, which is a significant concern^5^. Several alternative access sites for hemodialysis tunneled catheters have been documented, such as the infrarenal inferior vena cava through a translumbar approach^6^, intracardiac^5^, and transhepatic^5,7,8,9^. This study aimed to describe our center’s experience with transhepatic venous accesses for hemodialysis, focusing on the success rate of the implantation procedure, the incidence of complications, infection rates, catheter patency time, and the dialysis adequacy provided. Through the analysis of these data, we seek to assess the feasibility of using these accesses in hemodialysis patients with exhaustion of traditional vascular access locations.

## Methods

We conducted a retrospective observational study in which the clinical records of all patients who had a transhepatic CVC inserted or removed at *Pro-Rim* Foundation between January 2017 and February 2024 were reviewed. The patients’ clinical records were analyzed for demographic data, concomitant comorbidities, date of CVC placement and/or removal, indication for CVC placement, catheter patency time, dialysis adequacy (eKt/V), reason for CVC removal, patient survival, and cause of death. Primary patency time was defined as the time from catheter insertion to the first intervention due to dysfunction, while secondary patency was defined as the time from catheter insertion to permanent catheter removal, regardless of interventions. Complication rates, including infections and thrombosis, were also assessed. Catheter thrombosis was defined as the partial or complete obstruction of blood flow within the CVC, diagnosed based on clinical signs such as increased resistance to blood flow, inability to aspirate blood, or failure to achieve the prescribed dialysis blood flow rate.

**Figure 1:**
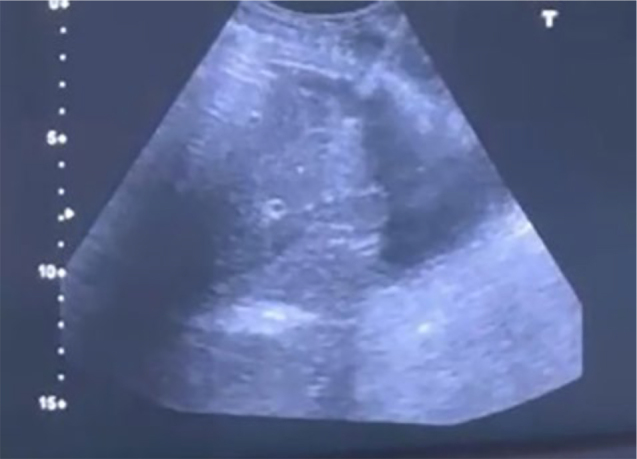
Hepatic vein puncture with a 21-gauge needle (ultrasound guidance image).

**Figure 2:**
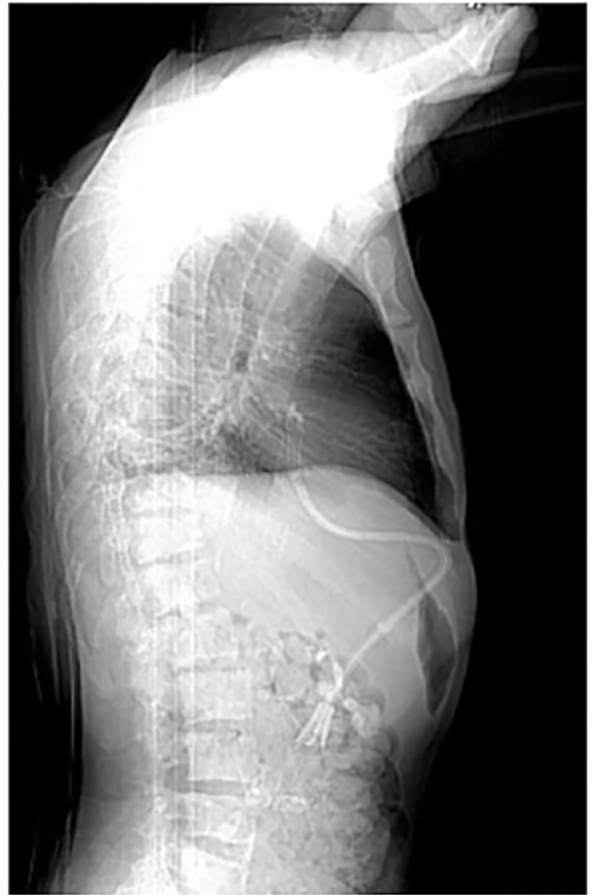
Post-procedure radiograph showing the positioning of the transhepatic hemodialysis catheter with its inner tip in the right atrium.

At our center, transhepatic catheters are placed with real-time ultrasound guidance and fluoroscopy. The procedure involves the introduction of a 21-gauge needle into the right hepatic vein through a subcostal or intercostal approach (Figure 1). Once contrast injection confirmed the appropriate position into the hepatic vein, a guidewire is passed through the needle into the inferior vena cava (IVC). The tract is dilated to accommodate a peel-away sheath. A subcutaneous tunnel is created inferiorly in the mid-axillary region, and the catheter is positioned with its inner tip in the right atrium (Figure 2). All patients were on a chronic hemodialysis program and could no longer have a venous access placed in a conventional location, nor did they have suitable vessels to construct an AVF.

## Results

Between January 2017 and February 2024, a total of 24 long-term transhepatic CVC were inserted in 12 patients at our center. Seven (58.3%) patients were male and five (41.6%) were female, with a mean age of 55.9 ± 16.7 years (minimum 33; maximum 87 years). The main cause of end-stage renal disease (ESRD) in these patients was diabetic kidney disease (n = 6; 50.0%). Three (25.0%) patients had ESRD of undetermined etiology. The remaining patients had ESRD in the context of cardiorenal syndrome, hypertensive nephrosclerosis and mesangioproliferative glomerulonephritis. Regarding concomitant comorbidities, 8 patients (66.7%) had arterial hypertension, 6 (50.0%) had diabetes mellitus, and 5 (41.7%) had heart failure, one of whom had reduced left ventricular ejection fraction. Two (16.7%) patients had a previous history of transplantation and six (50.0%) were currently on the kidney transplant waiting list.

Among the 24 long-term transhepatic venous access placed, 14 corresponded to new catheters implantations and 10 corresponded to replacements. Technical success was achieved in 100% of the cases and there were no complications in the first 24 hours after the procedure. The transhepatic CVC were in place for a total of 3615 catheter-days, with a mean primary catheter’s patency time of 162.9 days (minimum 1; maximum 690 days) and a secondary patency time of 204.0 days (minimum 12; maximum 601 days).

There were 6 early complications (defined as occurring within the first 30 days after implantation) and 8 late complications. The early complications observed were 5 catheter thrombosis and 1 catheter infection. On the other hand, the late complications described were 6 access thrombosis and 2 catheter infections. No migrations or displacements of the CVC were observed.

Ten catheters had to be replaced due to catheter thrombosis. It should be noted that four of these replacements were performed in the same patient, who was a 33-year-old diabetic woman with a prothrombotic coagulation disorder. In one case of access thrombosis, only catheter removal was performed, and replacement was not necessary because the patient was transferred to peritoneal dialysis. Catheter thrombosis rate was 0.30 per 100 catheter-days. Two catheters were removed due to infection: one case of sepsis and one case of CVC tunnel infection. The case of tunnel infection was of the previously mentioned woman who had the history of multiple access replacements because of thrombosis. In this case, the removed catheter had been in place for 29 days, and a new catheter was placed the same day due to an urgent need for dialysis, with the tunnel constructed at in a different location. Catheter infection complicated with sepsis occurred in a catheter which had been in place for 86 days. This access was removed and the new transhepatic CVC was implanted twelve days later, since the patient’s residual renal function allowed the placement of the catheter to be postponed. The infection rate was 0.08 per 100 catheter-days.

The mean dialysis dose (assessed by eKt/V) observed with these transhepatic CVC was 1.29 (minimum 0.80; maximum 1.77).

Seven patients died during the follow-up time, all with functioning catheters. Only one death was related to vascular access complications (sepsis). Four patients died of cardiovascular causes and in the remaining cases, the cause of death was unknown.

## Discussion

Improvements in hemodialysis therapy have extended the life expectancy of patients relying on this method of organ replacement^10^. The growing number of patients on hemodialysis and the increased survival of these patients^1,10^ rise the likelihood of thrombosis in conventional blood vessels used for central catheter placement^7^. The progressive lack of appropriate veins for creating AVF is also an important issue. Therefore, when an AVF cannot be created in time or when it is not possible to create it due to inadequate veins, the patient becomes dependent on a long-term CVC to undergo hemodialysis. The advantage of the internal jugular vein as a location for CVC implantation is widely known^2,3,4^ due to its easier approach and lower complication rate, being the recommended primary choice for long-term CVC placement according to KDOQI guidlines^2^. As mentioned, the internal jugular veins have low complication rates (stenosis/thrombosis), which can reach a maximum of 10%, while in the subclavian veins, these rates can be as high as 50%^3^. Furthermore, the implantation of a CVC in the internal jugular veins favors the preservation of the veins in the arms and legs for potential future grafts or AVF. It is worth noting that subclavian and femoral catheterization can compromise the entire limb for another hemodialysis access in case of stenosis or thrombosis^7^. On the other hand, kidney transplant is the preferred treatment for ESRD in selected patients^11^, which also requires the presence of an adequate vascular patrimony to receive the new organ, especially the iliac vessels. It should be noted that these vessels may be compromised by the previous presence of a CVC in the femoral veins.

In patients undergoing CVC-dependent hemodialysis who present an exhaustion of the traditional locations for CVC placement (such as jugular, subclavian and femoral veins), it is necessary to consider alternative access sites for hemodialysis tunneled catheters. The first case report of a transhepatic catheter for hemodialysis was made by Po et al.^12^ in 1994. Since then, other authors have published small retrospective studies describing the implantation of long-term percutaneous transhepatic catheters as an alternative access for hemodialysis in patients who no longer have conventional access sites available^5,7,8,9^.

The most frequently approached veins for transhepatic catheter implantation are the right and middle hepatic veins. The right hepatic vein is preferred due to its more peripheral location, longer path, and because it has a more horizontal upper portion in the direction of the inferior vena cava. In our center, all percutaneous transhepatic catheters were placed via the right hepatic vein approach.

Compared to other alternative vascular accesses such as the translumbar approach, the transhepatic catheterization seems to have a lower risk of damage and bleeding. Additionally, complications can typically be managed effectively through embolization of the liver parenchyma tract if needed. The transhepatic approach is often simpler than the translumbar approach, and can be successfully performed even when the lower segment of the inferior vena cava is completely occluded^13^. Revisions are simpler with this approach compared to the translumbar path, as fibrosis along the retroperitoneal tract can make the revision process more technically demanding^8^.

Stavropoulos et al., in their study about transhepatic catheters, reported that placing the catheter via the hepatic vein rather than directly into the inferior vena cava reduces the likelihood of catheter displacement and migration, considering the greater intravascular path^7^. In our study, no complications related to the migration of the transhepatic catheter were observed.

Only a few studies have examined patients with transhepatic catheters, which is understandable given this technique’s role as an alternative, lastresort option. In one study, Stavropoulos et al.^7^ evaluated 12 patients with a total of 32 transhepatic catheters. Likewise, Smith et al.^14^ assessed the safety and effectiveness of 21 transhepatic hemodialysis catheters in 16 patients. El Gharib et al.^8^ studied 23 patients who received 25 transhepatic catheters, while Motta-Leal-Filho et al.^9^ examined 6 patients with 9 catheters. Barros et al.^5^ described another small case series of 4 transhepatic catheters. These studies had small sample sizes, typical in this area of research. In our study, we reviewed 12 patients with a total of 24 transhepatic catheters (14 were new catheters implantations and 10 were replacements). Technical success was achieved in 100% of the cases and there were no complications in the first 24 hours after the procedure, as reported in previous studies^5,9,14^. These findings demonstrate that transhepatic central venous catheters for hemodialysis can be placed safely.

In our study the transhepatic catheters were in place for a total of 3615 catheter-days, with a mean primary and secondary catheter’s patency times of 162.9 days and 204.0 days, respectively. Regarding primary patency time, our results surpassed those reported by Stavropoulos et al.^7^ who observed a mean primary patency time of 27 catheter-days. However, they were inferior to those reported by other groups of researchers, such as El Gharib et al.^8^ who described a mean primary patency time of 210 catheter-days. The smaller sample size and the higher mean age of participants in our study may partially explain these findings.

In their research, Motta-Leal-Filho et al.^9^ reported two cases of catheter dislocation requiring repositioning and one case of complete migration outside the deep venous system, amounting to a 33% rate of displacement or migration. The authors attributed these events primarily to respiratory movements, which can push the catheter backward between the liver and the chest wall. Early detection of such complications is essential, making it critical to monitor the CVC closely during hemodialysis^9^. At the first sign of low flow, the catheter should be evaluated to identify potential migration, as this risk is higher with this type of access compared to CVC placed in traditional sites.

A large study of over 50,000 patients with CVC from the Strategic Healthcare Programs National Database (USA) show that thrombotic occlusions were responsible for 28% of catheter malfunctions, making it the most frequent complication^15^.

In their study, Stavropoulos et al.^7^ reported a high rate of transhepatic catheter thrombosis (2.4 per 100 catheter-days), which certainly contributed to the low primary patency times they observed. In contrast, our study demonstrated a significantly lower access thrombosis rate of 0.3 per 100 catheter-days. However, even lower rates have been documented in other research, with El Gharib et al.^8^ reporting a transhepatic catheter thrombosis rate of 0.13 per 100 catheter-days. Motta-Leal-Filho et al.^9^ reported a transhepatic catheter thrombosis rate of 0.05 per 100 catheter-days, which are data similar to those of other publications that used different types of venous access, such as the internal jugular vein, the femoral vein, and the translumbar access^16,17,18^. The consistent use of heparin after each hemodialysis session reduces the risk of developing thrombi and seems to be the main factor attributed to the better results regarding thrombosis access rate^8^. Furthermore, these findings highlight the close connection between thrombosis rate and primary patency time of the CVC, as thrombosis is one of the main causes of catheter malfunction^15^.

Our study reported a vascular access infection rate of 0.08 per 100 catheter-days, which is lower than the rates documented by Stavropoulos et al.^7^ and El Gharib et al.^8^ in their research (0.22 and 0.13 per 100 catheter-day, respectively). On the other hand, our infection rate was similar to that reported by Motta-Leal-Filho et al.^9^ in their study on transhepatic catheters and was also align with data from other studies examining different types of central venous access^16,17,18^.

Data on dialysis adequacy with transhepatic catheters is limited. Barros et al.^5^ reported eKt/V values ranging from 1.85 to 1.9 in their study. In comparison, our study observed a lower average dialysis dose (mean eKt/V of 1.29), but nevertheless a reasonable value for dialysis efficacy.

Hepatic tract embolization after elective catheter removal remains a topic of debate, with some authors saying that when a transhepatic CVC is removed, its path should be embolized^19^. In their revisions, Stavropoulos et al.^7^ and Motta-Leal-Filho et al.^9^ report that in all catheters removed, tract embolization with gelfoam pledgets was performed. On the other hand, in the work of Smith et al.^14^, the embolization was not routinely performed, and no increase in bleeding complications was observed among their patients. Similarly, in our study, none of the patients underwent tract embolization following catheter removal, and no bleeding complications were observed.

This study had some limitations. Firstly, it was a retrospective analysis that included a relatively small number of patients, which limits the generalizability of the findings. Additionally, there was no direct comparison with other nontraditional central venous access sites, which restricts the ability to evaluate the relative performance of transhepatic catheters against alternative approaches. Long-term follow-up was incomplete for seven patients, as they died during the study period while still utilizing functioning transhepatic catheters. Two of the five patients who were alive during follow-up time had the catheter implanted for less than 30 days, which restricted the number of patients with a longer followup time. These gaps in follow-up data may influence the overall assessment of long-term outcomes.

## Conclusion

Despite its limitations, our study supports some important conclusions about transhepatic hemodialysis catheters. This technique has demonstrated good long-term functionality and durability in patients who have exhausted all other traditional and alternative venous access options and are not candidates for peritoneal dialysis. The infection rates observed in this study were relatively low, suggesting that transhepatic catheter placement is a safe and viable option when other access sites are unavailable. The reasonable dialysis adequacy observed in our study also seems to support the viability of these accesses. However, thrombosis is a significant challenge in this type of access, as it is with venous access in other locations, making proper maintenance and close monitoring essential.

Overall, while transhepatic dialysis catheters should be regarded as a last-resort option, they provide a lifeline for patients in whom all other access options have been exhausted. Further research and advancements to reduce complications, such as thrombosis, could enhance the feasibility and outcomes of this technique, encouraging broader consideration in selected cases in clinical practice.

## Data Availability

The dataset supporting the results of this study is not publicly available.
