# Supplementary material for: Percutaneous transhepatic venous access for hemodialysis: a single-center experience with a rescue access
Source: J Bras Nefrol. 2026 Jan 30;48(1):e20240225. doi: 10.1590/2175-8239-JBN-2024-0225en (PMC12863402; doi:10.1590/2175-8239-JBN-2024-0225en)
Supplement: Translation

# Translation: Acesso venoso trans-hepático percutâneo para hemodiálise: experiência de centro único com acesso de resgate

**DOI:** 10.1590/2175-8239-JBN-2024-0225pt

**Authors:** Mariana Sousa Freitas, Amanda Meyer Luz, Ademar Regueira

**Affiliations:** 1 Centro Hospitalar de Trás-os-Montes e Alto Douro, Serviço de Nefrologia, Vila Real, Portugal.; 2 Hospital Regional Hans Dieter Schmidt, Serviço de Nefrologia, Joinville, SC, Brasil.; 3 Fundação Pró-Rim, Joinville, SC, Brasil.; 4 Hospital Municipal São José, Serviço de Nefrologia, Joinville, SC, Brasil.; 5 Hospital Municipal São José, Serviço de Cirurgia Vascular, Joinville, SC, Brasil.

**Keywords:** Cateteres Venosos Centrais, Hemodiálise, Cateter trans-hepático

## Abstract

**Introdução:**

Os cateteres venosos centrais (CVC) são frequentemente a única opção para hemodiálise, especialmente quando não é possível confeccionar fístulas arteriovenosas ou em situações de urgência. Entretanto, o esgotamento dos sítios de acesso tradicionais requer abordagens alternativas. Este estudo tem como objetivo descrever a experiência de nosso centro com acesso venoso transhepático para hemodiálise, focando nas taxas de infecção, patência do cateter e adequação da diálise para avaliar sua viabilidade em pacientes com opções limitadas de acesso vascular.

**Métodos:**

Estudo retrospectivo realizado na Fundação Pró-Rim (janeiro/2017 a fevereiro/2024) envolvendo pacientes com CVC trans-hepático. Analisaram-se registros clínicos quanto a dados demográficos, comorbidades, detalhes do CVC, adequação dialítica e desfechos.

**Resultados:**

24 CVC trans-hepáticos de longa permanência foram implantados em 12 pacientes (58,3% homens; idade média 55,9 anos). A taxa de sucesso técnico foi de 100%, sem complicações em 24 horas. Ao longo de 3615 dias-cateter, houve trombose a uma taxa de 0,30 por 100 dias-cateter e infecção a 0,08 por 100 dias-cateter. A dose média de diálise (eKt/V) foi 1,29. Sete pacientes morreram durante o acompanhamento, com apenas um óbito relacionado a complicações do acesso vascular. Tempos médios de patência primária e secundária dos cateteres foram 162,9 e 204,0 dias, respectivamente.

**Conclusão:**

Nosso estudo apoia o uso de cateteres trans-hepáticos para hemodiálise como opção viável para pacientes sem outras alternativas de acesso, demonstrando boa funcionalidade a longo prazo, baixas taxas de infecção e adequação dialítica razoável. A trombose permanece um desafio significativo, exigindo melhor manutenção, monitoramento e pesquisas adicionais para aprimorar os desfechos.

## Introdução

De acordo com o Censo Brasileiro de Diálise (CBD) da Sociedade Brasileira de Nefrologia (SBN)^1^, em julho de 2023, o número estimado de pacientes em diálise crônica era de 157.357, o que corresponde a um aumento de 2,3% em comparação ao ano anterior. Entre os pacientes prevalentes em diálise, 96,2% estavam em hemodiálise (HD) e 3,8% em diálise peritoneal (DP)^1^.

Para a realização da HD, é necessário criar um acesso vascular. A diretriz de prática clínica para acesso vascular da *Kidney Disease Outcomes Quality Initiative* (KDOQI) recomenda a fístula arteriovenosa (FAV) como o acesso de preferência, em vez do cateter venoso central (CVC), para a maioria dos pacientes incidentes e prevalentes em HD^2^. Esse acesso vascular autólogo requer planejamento e um período de maturação, não podendo ser confeccionado em muitos pacientes. Atualmente, durante as avaliações de mapeamento vascular, muitos indivíduos apresentam um patrimônio vascular deficiente, seja pela ausência de artérias e veias de calibre adequado – muitas vezes em decorrência de doenças crônicas, como diabetes e aterosclerose – seja por danos nas veias causados por diversas punções durante internações. Além disso, no Brasil, muitos pacientes são admitidos no pronto-socorro com doença renal crônica (DRC) em estágio avançado, exigindo o início urgente da diálise e, consequentemente, a necessidade de inserção de CVC, com a construção da FAV adiada para uma fase posterior.

Em julho de 2023, 29,0% das pessoas em hemodiálise crônica utilizavam um CVC de longa permanência como acesso vascular, de acordo com o CBD da SBN^1^. O patrimônio vascular deficiente mencionado anteriormente e o início urgente da diálise, sem tempo hábil para o planejamento do acesso vascular, justificam parcialmente esses números.

Pacientes que não podem ter uma FAV ou um enxerto arteriovenoso protético confeccionado devem permanecer em hemodiálise por meio de um CVC de longa permanência^2^. Diversos estudos demonstraram o benefício da veia jugular interna como primeira escolha para a inserção de cateteres de longa permanência, devido à facilidade da técnica e às baixas taxas de complicações^2,3,4^. No entanto, o uso crônico desses dispositivos acarreta riscos significativos, como infecções e falha no acesso vascular, com possível esgotamento dos sítios tradicionais para inserção do cateter, o que é uma preocupação relevante^5^. Foram documentados diversos sítios alternativos de acesso para cateteres tunelizados de hemodiálise, como a veia cava inferior infrarrenal por meio de uma abordagem translombar^6^, intracardíaca^5^ e trans-hepática^5,7,8,9^. Este estudo teve como objetivo descrever a experiência de nosso centro com acessos venosos trans-hepáticos para hemodiálise, com foco na taxa de sucesso do procedimento de implante, na incidência de complicações, nas taxas de infecção, no tempo de patência do cateter e na adequação dialítica proporcionada. Por meio da análise desses dados, buscamos avaliar a viabilidade do uso desses acessos em pacientes em hemodiálise com esgotamento dos locais tradicionais de acesso vascular.

## Métodos

Realizamos um estudo observacional retrospectivo no qual foram revisados os prontuários clínicos de todos os pacientes que tiveram um CVC transhepático inserido ou removido na Fundação Pró-Rim entre janeiro de 2017 e fevereiro de 2024. Os prontuários dos pacientes foram analisados quanto a dados demográficos, comorbidades concomitantes, data de inserção e/ou remoção do CVC, indicação para inserção do CVC, tempo de patência do cateter, adequação da diálise (eKt/V), motivo da remoção do CVC, sobrevida do paciente e causa do óbito. O tempo de patência primária foi definido como o intervalo entre a inserção do cateter e a primeira intervenção decorrente de disfunção, enquanto a patência secundária foi definida como o intervalo entre a inserção do cateter e sua remoção permanente, independentemente das intervenções. As taxas de complicações, incluindo infecções e trombose, também foram avaliadas. A trombose do cateter foi definida como a obstrução parcial ou completa do fluxo sanguíneo dentro do CVC, diagnosticada com base em sinais clínicos, como aumento da resistência ao fluxo sanguíneo, incapacidade de aspirar sangue ou falha em alcançar a taxa de fluxo sanguíneo da diálise prescrita.

Em nosso centro, os cateteres trans-hepáticos são inseridos com orientação por ultrassom em tempo real e fluoroscopia. O procedimento envolve a introdução de uma agulha de calibre 21 na veia hepática direita por meio de uma abordagem subcostal ou intercostal (Figura 1). Uma vez que a injeção de contraste confirmou a posição adequada na veia hepática, um fio-guia é conduzido através da agulha até a veia cava inferior (VCI). O trajeto é dilatado para acomodar uma bainha removível. A criação de um túnel subcutâneo é efetuada inferiormente na região médio-axilar. Posteriormente, o cateter é posicionado com sua extremidade interna no átrio direito (Figura 2). Todos os pacientes encontravamse submetidos a um programa de hemodiálise crônica e já não era viável obter acesso venoso em local convencional, tampouco possuíam vasos adequados para a construção de uma FAV.

**Figura 1:** Punção da veia hepática com agulha de calibre 21 (imagem guiada por ultrassonografia)

**Figura 2:** Radiografia pós-procedimento mostrando o posicionamento do cateter trans-hepático de hemodiálise com sua extremidade interna no átrio direito.

## Resultados

Entre janeiro de 2017 e fevereiro de 2024, um total de 24 CVC trans-hepáticos de longa permanência foram inseridos em 12 pacientes em nosso centro. Sete (58,3%) indivíduos eram do sexo masculino e cinco (41,6%) do sexo feminino, com idade média de 55,9 ± 16,7 anos (mínimo 33; máximo 87 anos). A principal causa de doença renal em estágio terminal (DRET) nesses pacientes foi a doença renal diabética (n = 6; 50,0%). Três sujeitos (25,0%) apresentaram DRET de etiologia indeterminada. Os demais pacientes tinham DRET no contexto de síndrome cardiorrenal, nefroesclerose hipertensiva e glomerulonefrite mesangioproliferativa. Em relação às comorbidades concomitantes, 8 indivíduos (66,7%) apresentaram hipertensão arterial, 6 (50,0%) tinham diabetes mellitus e 5 (41,7%) apresentaram insuficiência cardíaca, sendo que um deles apresentou fração de ejeção do ventrículo esquerdo reduzida. Dois pacientes (16,7%) tinham histórico prévio de transplante e seis (50,0%) encontravam-se atualmente na lista de espera para transplante renal.

Entre os 24 acessos venosos trans-hepáticos de longa permanência implantados, 14 corresponderam a novas inserções de cateteres e 10 a substituições. O sucesso técnico foi alcançado em 100% dos casos e não houve complicações nas primeiras 24 horas após o procedimento. Os CVC trans-hepáticos permaneceram em uso por um total de 3.615 dias-cateter, com um tempo médio de patência primária do cateter de 162,9 dias (mínimo de 1; máximo de 690 dias) e um tempo de patência secundária de 204,0 dias (mínimo de 12; máximo de 601 dias).

Houve 6 complicações precoces (definidas como aquelas ocorridas nos primeiros 30 dias após a implantação) e 8 complicações tardias. As complicações precoces observadas foram 5 tromboses e 1 infecção relacionada ao cateter. Por outro lado, as complicações tardias descritas foram 6 tromboses de acesso e 2 infecções relacionadas ao cateter. Não foram observadas migrações ou deslocamentos do CVC.

Dez cateteres precisaram ser substituídos devido à trombose do cateter. É importante ressaltar que quatro dessas substituições foram realizadas no mesmo paciente, uma mulher diabética de 33 anos com um distúrbio de coagulação pró-trombótico. Em um caso de trombose do acesso, apenas a remoção do cateter foi realizada, não sendo necessária a substituição, uma vez que o paciente foi transferido para diálise peritoneal. A taxa de trombose do cateter foi de 0,30 por 100 dias-cateter. Dois cateteres foram removidos devido à infecção: um caso de sepse e um caso de infecção do túnel do CVC. O caso de infecção do túnel ocorreu na paciente mencionada anteriormente, que apresentava histórico de múltiplas substituições de acesso devido à trombose. Nesse caso, o cateter removido estava em uso havia 29 dias, e um novo cateter foi implantado no mesmo dia devido à necessidade urgente de diálise, com o túnel confeccionado em um local diferente. A infecção do cateter complicada por sepse ocorreu em um cateter que estava em uso há 86 dias. Esse acesso foi removido e o novo CVC trans-hepático foi implantado doze dias depois, uma vez que a função renal residual do paciente permitiu que a inserção do cateter fosse adiada. A taxa de infecção foi de 0,08 por 100 dias-cateter.

A dose média de diálise (avaliada por eKt/V) observada com esses CVC trans-hepáticos foi de 1,29 (mínimo 0,80; máximo 1,77).

Sete pacientes foram a óbito durante o período de acompanhamento, todos com cateteres em funcionamento. Apenas um óbito foi relacionado a complicações do acesso vascular (sepse). Quatro pacientes faleceram por causas cardiovasculares e, nos demais casos, a causa do óbito foi desconhecida.

## Discussão

Os avanços na terapia de hemodiálise têm prolongado a expectativa de vida dos pacientes que dependem desse método de substituição de órgãos^10^. O número crescente de indivíduos em hemodiálise e o aumento da sobrevida desses pacientes^1,10^ aumentam a probabilidade de trombose nos vasos sanguíneos convencionais utilizados para a inserção de cateteres centrais^7^. A perda progressiva de veias adequadas para a criação de FAV também constitui uma questão importante. Portanto, quando uma FAV não pode ser confeccionada a tempo ou quando não é possível criá-la devido à inadequação das veias, o paciente torna-se dependente de um CVC de longa permanência para realizar a hemodiálise. A vantagem da veia jugular interna como sítio para implante de CVC é amplamente conhecida^2,3,4^, devido à sua abordagem mais fácil e menor taxa de complicações, sendo a escolha primária recomendada para a colocação de CVC de longa permanência segundo as diretrizes da KDOQI^2^. Conforme anteriormente referido, as veias jugulares internas apresentam taxas de complicações (estenose/trombose) reduzidas, que podem atingir um máximo de 10%. Em contraste, nas veias subclávias, essas taxas podem chegar a 50%^3^. Além disso, a inserção de um CVC nas veias jugulares internas favorece a preservação das veias dos membros superiores e inferiores para futuros enxertos ou FAV em potencial. É importante ressaltar que a cateterização das veias subclávia e femoral pode comprometer todo o membro para outro acesso de hemodiálise em caso de estenose ou trombose^7^. Por outro lado, o transplante renal é o tratamento de escolha para a DRET em pacientes selecionados^11^, o que também requer a presença de um patrimônio vascular adequado para receber o novo órgão, especialmente os vasos ilíacos. É importante ressaltar que esses vasos podem estar comprometidos pela presença prévia de um CVC nas veias femorais.

Em pacientes submetidos à hemodiálise dependente de CVC que apresentam esgotamento dos locais tradicionais para colocação do acesso (como veias jugular, subclávia e femoral), torna-se necessário considerar sítios de acesso alternativos para cateteres tunelizados de hemodiálise. O primeiro relato de caso de um cateter trans-hepático para hemodiálise foi apresentado por Po et al.^12^ em 1994. Desde então, outros autores têm publicado pequenos estudos retrospectivos descrevendo a implantação de cateteres trans-hepáticos percutâneos de longa permanência como um acesso alternativo para hemodiálise em pacientes que já não dispõem de sítios convencionais^5,7,8,9^.

As veias mais frequentemente abordadas para a implantação de cateteres trans-hepáticos são a veia hepática direita e a veia hepática média. A veia hepática direita é preferida devido à sua localização mais periférica, trajeto mais longo e por apresentar uma porção superior mais horizontal na direção da veia cava inferior. Em nosso centro, todos os cateteres trans-hepáticos percutâneos foram implantados por meio da abordagem da veia hepática direita.

Em comparação com outros acessos vasculares alternativos, como a abordagem translombar, a cateterização trans-hepática parece apresentar um menor risco de danos e sangramento. Além disso, as complicações podem, normalmente, ser manejadas de forma eficaz por meio da embolização do trajeto do parênquima hepático, se necessário. A abordagem trans-hepática é frequentemente mais simples do que a translombar e pode ser realizada com sucesso mesmo quando o segmento inferior da veia cava inferior se encontra completamente ocluído^13^. Esta abordagem simplifica o procedimento de revisão em comparação com a via translombar, uma vez que a fibrose ao longo do trajeto retroperitoneal pode tornar o processo de revisão mais exigente tecnicamente^8^.

Stavropoulos et al., em seu estudo sobre cateteres trans-hepáticos, relataram que a inserção do cateter pela veia hepática, em vez de diretamente na veia cava inferior, reduz a probabilidade de deslocamento e migração do dispositivo, considerando o maior trajeto intravascular^7^. Em nosso estudo, não foram observadas complicações relacionadas à migração do cateter trans-hepático.

Apenas alguns trabalhos analisaram pacientes com cateteres trans-hepáticos, o que é compreensível, dado o papel dessa técnica como opção alternativa de último recurso. Em um dos estudos, Stavropoulos et al.^7^ avaliaram 12 pacientes com um total de 32 cateteres trans-hepáticos. Da mesma forma, Smith et al.^14^ analisaram a segurança e a eficácia de 21 cateteres trans-hepáticos para hemodiálise em 16 pacientes. El Gharib et al.^8^ estudaram 23 pacientes que receberam 25 cateteres trans-hepáticos, enquanto Motta-Leal-Filho et al.^9^ analisaram 6 pacientes com 9 cateteres. Barros et al.^5^ descreveram outra pequena série de casos composta por 4 cateteres trans-hepáticos. Esses estudos apresentaram amostras de pequeno porte, típico dessa área de pesquisa. Em nosso estudo, revisamos 12 pacientes com um total de 24 cateteres trans-hepáticos (14 novas implantações e 10 substituições). O sucesso técnico foi alcançado em 100% dos casos e não houve complicações nas primeiras 24 horas após o procedimento, conforme relatado em estudos anteriores^5,9,14^. Esses achados demonstram que os cateteres venosos centrais trans-hepáticos para hemodiálise podem ser implantados com segurança.

Em nosso estudo, os cateteres trans-hepáticos permaneceram em uso por um total de 3.615 dias-cateter, com tempos médios de patência primária e secundária do cateter de 162,9 dias e 204,0 dias, respectivamente. Em relação ao tempo de patência primária, nossos resultados superaram os relatados por Stavropoulos et al.^7^ que observaram um tempo médio de patência primária de 27 dias-cateter. No entanto, eles foram inferiores aos relatados por outros grupos de pesquisadores, como El Gharib et al.^8^, que descreveram um tempo médio de patência primária de 210 dias-cateter. O tamanho reduzido da amostra e a idade média mais elevada dos participantes em nosso estudo podem explicar parcialmente esses achados.

Em sua pesquisa, Motta-Leal-Filho et al.^9^ relataram dois casos de deslocamento do cateter que exigiram reposicionamento e um caso de migração completa para fora do sistema venoso profundo, totalizando uma taxa de 33% de deslocamento ou migração. Os autores atribuíram esses eventos principalmente aos movimentos respiratórios, que podem empurrar o cateter para trás, entre o fígado e a parede torácica. A detecção precoce dessas complicações é essencial, tornando fundamental o monitoramento rigoroso do CVC durante a hemodiálise^9^. Ao primeiro sinal de baixo fluxo, o cateter deve ser avaliado para identificar uma possível migração, uma vez que esse risco é maior com esse tipo de acesso em comparação ao CVC inserido em sítios tradicionais.

Um amplo estudo envolvendo mais de 50.000 pacientes com CVC, proveniente do *Strategic Healthcare Programs National Database* (EUA), demonstrou que as oclusões trombóticas foram responsáveis por 28% das disfunções do cateter, tornando-se a complicação mais frequente^15^.

Em seu estudo, Stavropoulos et al.^7^ relataram uma taxa elevada de trombose relacionada ao cateter trans-hepático (2,4 por 100 dias-cateter), o que certamente contribuiu para os baixos tempos de patência primária observados. Em contraste, nosso estudo demonstrou uma taxa significativamente menor de trombose de acesso, de 0,3 por 100 dias-cateter. No entanto, taxas ainda mais baixas foram documentadas em outras pesquisas, como a de El Gharib et al.^8^ que relatou uma taxa de trombose de cateter trans-hepático de 0,13 por 100 dias-cateter. Motta-Leal-Filho et al.^9^ relataram uma taxa de trombose de cateter trans-hepático de 0,05 por 100 dias-cateter, dados semelhantes aos de outras publicações que utilizaram diferentes tipos de acesso venoso, como a veia jugular interna, a veia femoral e o acesso translombar^16,17,18^. O uso consistente de heparina após cada sessão de hemodiálise reduz o risco de desenvolvimento de trombos e parece ser o principal fator atribuído aos melhores resultados quanto à taxa de trombose de acesso^8^. Além disso, esses achados destacam a estreita ligação entre a taxa de trombose e o tempo de patência primária do CVC, uma vez que a trombose é uma das principais causas do funcionamento inadequado do cateter^15^.

Nosso estudo relatou uma taxa de infecção do acesso vascular de 0,08 por 100 dias-cateter, valor inferior às taxas documentadas por Stavropoulos et al.^7^ e El Gharib et al.^8^ em suas pesquisas (0,22 e 0,13 por 100 dias-cateter, respectivamente). Por outro lado, nossa taxa de infecção foi semelhante à relatada por Motta-Leal-Filho et al.^9^ em seu estudo sobre cateteres trans-hepáticos e também esteve alinhada com os dados de outros trabalhos que analisaram diferentes tipos de acesso venoso central^16,17,18^.

Os dados sobre a adequação da diálise com cateteres trans-hepáticos são limitados. Barros et al.^5^ relataram valores de eKt/V variando de 1,85 a 1,9 em seu estudo. Em comparação, nosso trabalho observou uma dose média de diálise mais baixa (média de eKt/V de 1,29), mas, ainda assim, um valor razoável para a eficácia da terapia.

A embolização do trajeto hepático após a remoção eletiva do cateter permanece um tema de debate, com alguns autores afirmando que, quando um CVC transhepático é removido, a via de acesso deve ser embolizada^19^. Em suas revisões, Stavropoulos et al.^7^ e Motta-Leal-Filho et al.^9^ relataram que, em todos os cateteres removidos, foi realizada embolização do trajeto com *pledgets* de *Gelfoam*. Por outro lado, no trabalho de Smith et al.^14^, a embolização não foi realizada de forma rotineira, e não observou-se aumento nas complicações hemorrágicas entre seus pacientes. De forma semelhante, em nosso estudo, nenhum dos pacientes foi submetido à embolização do trajeto após a remoção do cateter, e não foram observadas complicações hemorrágicas.

Este estudo apresentou algumas limitações. Em primeiro lugar, foi uma análise retrospectiva que incluiu um número relativamente pequeno de pacientes, o que limita a generalização dos achados. Além disso, não houve comparação direta com outros sítios de acesso venoso central não tradicionais, o que restringe a capacidade de avaliar o desempenho relativo dos cateteres transhepáticos em relação a abordagens alternativas. O acompanhamento de longo prazo foi incompleto para sete pacientes, uma vez que eles foram a óbito durante o período do estudo enquanto ainda possuíam cateteres trans-hepáticos em funcionamento. Dois dos cinco pacientes que permaneceram vivos durante o período de acompanhamento tiveram o cateter implantado por menos de 30 dias, o que restringiu o número de participantes com um período de acompanhamento mais prolongado. Essas lacunas nos dados de acompanhamento podem influenciar a avaliação geral dos desfechos de longo prazo.

## Conclusões

Apesar de suas limitações, nosso estudo corrobora algumas conclusões importantes sobre os cateteres trans-hepáticos para hemodiálise. Essa técnica demonstrou boa funcionalidade e durabilidade a longo prazo em pacientes que exauriram todas as demais opções tradicionais e alternativas de acesso venoso e não são candidatos à diálise peritoneal. As taxas de infecção observadas neste estudo foram relativamente baixas, sugerindo que a inserção de cateter trans-hepático é uma opção segura e viável quando outros sítios de acesso não estão disponíveis. A adequação razoável da diálise observada em nosso estudo também parece apoiar a viabilidade desses acessos. No entanto, a trombose constitui um desafio significativo nesse tipo de acesso, assim como no acesso venoso em outros sítios, tornando essencial a manutenção adequada e o monitoramento rigoroso.

De modo geral, embora os cateteres transhepáticos para diálise devam ser considerados uma opção de último recurso, eles representam uma alternativa vital para pacientes nos quais todas as demais opções de acesso foram esgotadas. Pesquisas adicionais e avanços no sentido de reduzir complicações, como a trombose, podem aprimorar a viabilidade e os desfechos dessa técnica, incentivando uma consideração mais ampla em casos selecionados na prática clínica.

## Data Availability

O conjunto de dados que sustenta os resultados deste estudo não está disponível publicamente.
